# Dr. Adams, on Herpes during Vaccination

**Published:** 1806-09

**Authors:** Joseph Adams

**Affiliations:** Berner's Street


### *To the Editors of the Medical and Physical Journal.*

Gentlemen,

It has been a common, and certainly a too just complaint against your Journal, that the expressions in some controversial papers are much too severe. I am ready to admit, that, when two gentlemen expose themselves by the warmth with which they conduct their disputes, there are others who will feel gratified and amused at their expense. But, however entertaining this may be to the reader at first; a dispute managed without politeness soon becomes irksome; and such of the disputants as think the best and feel the most as they ought to do, will be the earliest at resigning the contest. So it is when two hackney coachman or two fishwomen quarrel: the bystanders are entertained, till at last vulgarity loses its force by losing its novelty. But if either of this description quarrels with one better bred, the latter instantly finds he is no match for one who trusts not to the justice of his cause, but to his recollection from the vocabulary of abuse. In consequence of this, the successful champion, fancying it is enough to vanquish his adversary, grows bold in proportion to his supposed prowess, till one, as brutal as himself, and with a stronger voice, convinces him that he also is vulnerable. It is true, this is not often the case; because, as "out of the abundance of the heart the mouth speaketh," so in general we find, that those who are thus indiscriminate in abuse, are composed of materials insensible to reproof of any kind.

This may, perhaps, be called a high charged picture; but it is on that account the more agreeable, to reflect how much your Journal is improved in so important a point, as may be seen by the manner in which a few papers have passed between some of your correspondents on the interruption to vaccination from herpetic blotches. Dr. Jenner's paper goes to show, that the interruption is various and uncertain, depending, among other things, on the period that the herpetic disposition has influenced the constitution, and that when such a state of the skin has become inveterate, it will sometimes be superseded by vaccination; in which case the latter will preserve its proper character. Mr. Clement and Dr. Wood have both afforded some very useful papers on the subject, and both have confirmed Dr.

Jenner's observations,* and the remarks I ventured to make on the same, in the subsequent number of your Journal. † Dr. Jenner observes, that if the skin is affected by any form of herpes, vaccination will be either altogether prevented by it, or that if vaccination succeeds, it will alter the form and character of the former eruption, which will now partake of the vaccine character, and probably scab and disappear with it. Mr. Clement gives several good cases of the former, and Dr. Wood of the latter. Both the papers are equally valuable in these respects. Dr. Wood, in your number for July, gives a striking proof of one part of Dr. Jenner's observations: "Herpes," says he (page 68, No. 89,) *of very long standing*, did not in the least prevent the infection of cow-pox taking place, nor affect the appearance of the pustule. The constitutional action induced by cow-pox tends to remove the herpetic disease." Now, nothing can be a stronger proof of the truth of Dr. Jenner's position. "I do not mean," says that gentleman, "that the pustule is always imperfect, and not effective when the inoculated patient has this malady: on the contrary, it is sometimes perfectly correct, and much more frequently so when it has been *of long standing*, than when in its recent state; and what is remarkable, the disease is then, (when of long duration) sometimes swept entirely away."—These events, as I observed in the paper above alluded to, are perfectly consistent with what happens in many other constitutional complaints, which at one time prevent, and at others are superseded by, different morbid poisons.

The name of Dr. Wood reminds me of his former paper, in which he expresses a claim the public have on me, as physician to the Small-pox Hospital. That claim I am ready to admit; and from the moment I was thought worthy of such an office, no industry on my part has been wanting in the examination of that invaluable prophylactic which we owe to Dr. Jenner, and also of a disease, which, I trust, will hereafter be only known in books. —The epidemic small-pox of last year has furnished every practical trial that can be suggested. Dr. Wood's remark would not so long have remained unnoticed, had it been as new as he suspected. In the early stages of small-pox, however confluent, the antiphlogistic plan has been regularly pursued ever since the Suttons revived it. But the *confluent* disease should not be confounded with

* See Journal Vol. XII. p. 97. † Ibid. 194.

with the *sessile* accompanied with purple spots and extravasations of blood. These cases are attended with such instant and universal prostration of strength, that no one has had the hardiness to propose free evacutions of any kind. The general fatality of the disease in this form, might indeed authorize some boldness of practice, were it not that spontaneous hæmorrhage or diarrhœa is always found to accelerate the close of the tragedy.

The cold treatment, with early evacuations, in confluent small-pox, seems to have been first introduced by Sydenham; at least, it is not easy to trace it further back. But it appeared so much at variance with his theory of despumation, and of the necessity of dispelling the morbific matter by the skin, that his own admirers were among the first to abandon a practice, which nothing but the closeness of his observation would have induced him to employ. Mead, who was an advocate for purgatives, and rested his practice on the authority of Sydenham, with whom he was contemporary, confined his patients to bed in the early stage of the eruption.

It may seem strange, in a disease in which external cold is so serviceable, that the occasional use of the tepid bath should be found a most valuable remedy. Wherever there appears local conjestion, or strong vascular action, confined to an important organ, the tepid bath seems to act like a charm. In delirium, it has several times produced instant relief; and as far as could be judged by the event, has not exasperated the future progress of the disease.

Thus far I have trespassed on your sheets, that I might not appear inattentive to Dr. Wood's suggestions. In a few weeks I hope to offer the public the result of my remarks on this disease; on its analogy in some instances with the cow-pox, whilst it constantly preserves its own characteristic distinctions; on the nice shades which separate the two; and also the chicken-pox, and other exanthemata which, in their more violent forms, approach so nearly to the mild forms of small-pox. One remark, however, may be allowed me. Having on a former occasion acknowledged that I could not, in several instances, discover the characteristic appearance of the slough in small-pox, you will suppose I have not been backward in tracing the cause of this variety. It depends much on the progress of the disease, and the period at which the pustule is dissected. For some time past, since Mr. Wachsel and myself have had leisure to renew the search, we have not met with an instance in which the slough has not been easily easily removed by the forceps (without destroying its figure) from the bottom of the foveola. I believe it will be found that wherever the bottom is sloughy, which is constant in most species of small-pox, that the slough may always be removed at a late period from the extremities where the suppuration is gradual, and the form of the pustule uninterrupted by early scabbing. I should not have troubled you on a subject which will soon appear before the public with a minuteness of detail inadmissable in your Journal, but as all this has reference to what has already been inserted by you, so much respect seemed due to your readers, and to the attention you have paid my former communications. I am, &c.

*Berner's Street, August* 15, 1806. JOSEPH ADAMS.

